# Analysis on efficacy of magnetic resonance lymphangiography using INV-001 in healthy beagle dogs

**DOI:** 10.1038/s41598-024-61104-4

**Published:** 2024-05-07

**Authors:** Ji Sung Jang, Nari Kim, Mi-Hyun Kim, Do-Wan Lee, Ji-wook Kim, Tae-Hyun Shin, Hyo Jung Park, Kyung Won Kim

**Affiliations:** 1 Departments of Radiology and Research Institute of Radiology, Asan Medical Center, College of Medicine, University of Ulsan, Olymphic-ro 43 Gil 88, Songpa-gu, Seoul, 138-735 Republic of Korea; 2 Departments of Medical Science, Asan Medical Institute of Convergence Science and Technology, Asan Medical Center, University of Ulsan College of Medicine, Seoul, Republic of Korea; 3 Research Institute, Trial Informatics Incorporated, Seoul, Republic of Korea; 4 Inventera Incorporated, Seoul, Republic of Korea; 5 Department of Radiation Science and Technology, Jeonbuk National University, Jeonju, Republic of Korea

**Keywords:** Medical research, Materials science

## Abstract

We aimed to conduct a proof-of-concept study of INV-001 in visualizing lymphatic vessels and nodes without venous contamination and to determine the optimal dose condition of INV-001 for magnetic resonance lymphangiography (MRL) in healthy beagles. MRL was performed using a 3.0-Tesla (T) whole body clinical magnetic resonance imaging (MRI) scanner. A dose-finding study of INV-001 for MRL in beagles (N = 6) was carried out according to an adaptive optimal dose finding design. For the reproducibility study (N = 6), MRL was conducted at selected INV-001 doses (0.056 and 0.112 mg Fe/kg) with a 15 mM concentration. Additionally, an excretion study (N = 3) of INV-001 was conducted by analyzing T_1_, T_2_, and T_2_* maps of the liver and kidney 48 h post-administration. INV-001 administration at doses of 0.056 and 0.112 mg Fe/kg (concentration: 15 mM) consistently demonstrated the visualization of contrast-enhanced lymphatic vessels and nodes without venous contamination in the beagles. The contrast enhancement effect was highest at 30 min after INV-001 administration, then gradually decreasing. No toxicity-related issues were identified during the study. After 48 h, the T_1_, T_2_, and T_2_* values in the liver and both kidneys were found to be comparable to the pre-administration values, indicating thorough INV-001 excretion. The optimal dosing conditions of INV-001 for MRL for contrast-enhanced visualization of lymphatic vessels and nodes exclusively with no venous contamination in beagles was determined to be 0.056 mg Fe/kg with a 15 mM concentration.

## Introduction

Lymphedema is the result of the interstitial accumulation of lymphatic fluid due to damage to the lymphatic vessel^1,2^. The imaging of lymphedema is performed when lymphedema is not clear or a more definitive diagnosis is needed considering the prognosis and treatment. However, lymphatic vessels are 0.4–0.8 mm in diameter, almost 10 times smaller than the blood vasculature, making it difficult to directly visualize the lymphatic system^3^. To address this difficulty, magnetic resonance (MR) lymphangiography (MRL) using contrast agents have been widely used in clinical practice to visualize the lymphatic vessels and lymphedema with high resolution anatomical detail and soft tissue contrast^3–7^.

**Figure 1:**
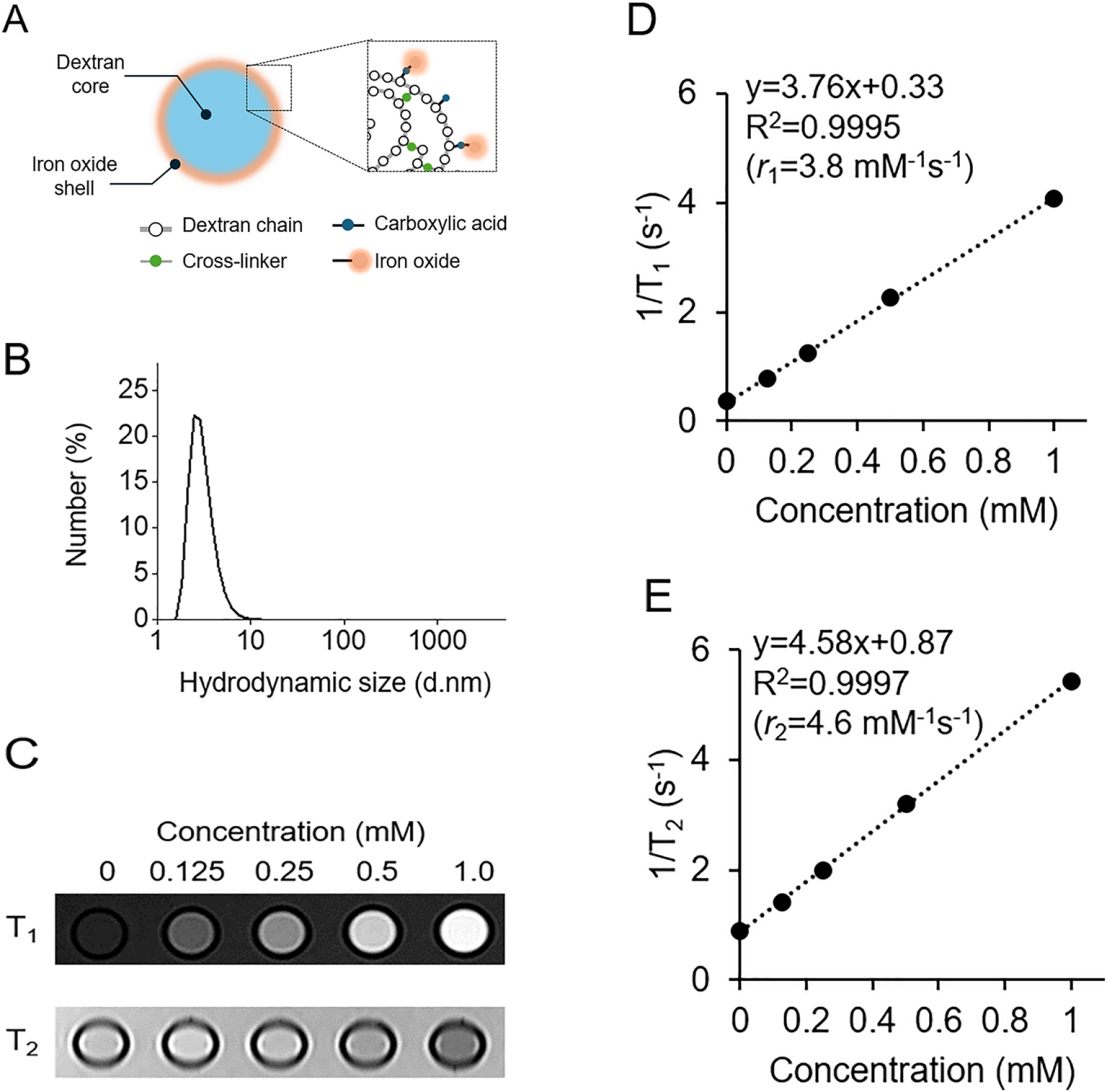
Characterization of INV-001. (**A**) The structure of INV-001 comprises a dextran core and an iron oxide shell. (**B**) Hydrodynamic size of INV-001. (**C**) T_1_- (top) and T_2_- (bottom) weighted MR images of the INV-001 phantom with concentration of 0, 0.125, 0.25, 0.5, and 1.0 mM. (**D**,**E**) Plots of 1/T_1_ (**D**) and 1/T_2_ (**E**) versus iron concentration of INV-001 for T_1_ and T_2_ relaxivity coefficients (*r*_1_ and *r*_2_) calculation.

Gadolinium-based contrast agents (GBCAs) are currently used for MRL^6,8–10^. However, the use of GBCAs induce visualization of both lymphatic vessels and normal veins^9,11–14^. The normal veins often obscure the lymphatic vessels and impair the diagnostic value of MRL; this is called venous contamination. Some studies have highlighted the issue with small molecular sizes of gadolinium-based contrast agents (GBCAs) facilitates non-selective diffusion into the venous system, which has been associated with venous contamination problems^3,15^. These interferences not only compromise the diagnostic accuracy of MRL but also heighten the risk of misinterpreting imaging results, which could culminate in diagnostic inaccuracies. To overcome this, a new type of T_1_ contrast agent, which consists of dextran core-iron oxide shell (Fig. 1A, INV-001, Inventera Inc., Seoul, Republic of Korea), was developed^16^. Due to its small hydrodynamic size (3.6 nm, Fig. 1B) and dextran core-iron oxide shell structure, INV-001 exhibits a very small *r*_2_/*r*_1_ value, an important parameter for a T_1_ contrast agent (optimal value: ~ 1), with a value of 1.2. Since the hydrodynamics size of INV-001 is 3.6 nm which may be relatively large for intravasation, intradermally/subcutaneously injected INV-001, is assumed to selectively taken up by lymphatic vessel, thereby visualize only lymphatic vessels without venous contamination^17^.

The purpose of this study was to validate the efficacy of INV-001 in visualizing lymphatic vessels without venous contamination. We also aimed to determine the optimal dosing condition of INV-001 for MRL in beagle dogs.

## Materials and methods

### Preparation of INV-001

INV-001 was synthesized using the following procedure. Briefly, a 20 mM solution of Dextran T5 (5510 0005 9006, Pharmacosmos Inc., Watchung, NJ, USA) was mixed with a sodium hydroxide solution and epichlorohydrin (45,340, Sigma-Aldrich, St. Louis, MO, USA), followed by the addition of diethylenetriamine (D93856, Sigma-Aldrich, St. Louis, MO, USA). The cross-linked dextran was then purified using a 3-kDa cut-off ultrafiltration filter (UFC900308, Millipore, Bedford, MA, USA). Subsequently, the terminal amine was modified into a carboxyl functional group. After ultrafiltration, the carboxyl-functionalized dextran was reacted with an iron chloride solution under basic pH condition for 1 h. Finally, the resulting products were purified and concentrated using ultrafiltration.

### Hydrodynamic size measurement of INV-001

The hydrodynamic size was measured using a dynamic light scattering (DLS, Zetasizer Pro, Malvern Panalytical Ltd, Malvern, UK) device with the following parameters: a reflective index of 1.48 and an absorption of 0.001.

### MRI Phantom study of INV-001

MRI Phantom images were acquired with a 3.0 Tesla (T) magnet machine (MAGNETOM Vida 3 T, Siemens Healthcare, Erlangen, Germany). INV-001 with concentration of 0.125, 0.25, 0.5, and 1.0 mM were prepared in sealed tubes filled with distilled water to minimize image artifacts. To obtain the MR relaxivities (*r*_1_ and *r*_2_) of the INV-001, T_1_ (1) and T_2_ (2) mapping were performed as follows: (1) 8 inversion times (TI) = [20, 100, 200, 400, 800, 1000, 2000, 4000] ms, repetition time (TR) = 4900 ms, echo time (TE) = 7 ms, average = 1, slice thickness = 1.0 mm, matrix size = 96 × 96, and field of view (FOV) = 70 × 60 mm^2^. (2) 15 TEs = [10, 20, 30, 40, 50, 60, 70, 80, 90, 100, 110, 120, 130, 140, 150] ms, TR = 3000 ms, average = 1, slice thickness = 1.0 mm, matrix size = 96 × 96, and field of view = 70 × 60 mm^2^. T_1_ and T_2_ values were analyzed by drawing regions of interest (ROI) in different tubes for the analysis.

### Toxicity study of INV-001

Animal experiments were conducted in accordance with the Animal Research: Reporting of In Vivo Experiments (ARRIVE) guidelines and regulations of the Institutional Animal Care and Use Committee (IACUC) of Biotoxtech Co., Ltd. (Approval No. 220615). Additionally, all procedures in this study adhered to the Animal Protection Act of the Republic of Korea and the Guide for the Care and Use of Laboratory Animals. INV-001 was administered via intradermal injection once every 2 weeks (a total of three times) to beagles of both sexes for 4 weeks. The test groups consisted of three INV-001 dosing groups at 1.56, 3.12, and 6.24 mg Fe/kg and one control group (saline), with three animals of each sex per group.

### Animals

In this study, 2-y-old healthy male beagles (n = 3) that were 9.3 to 10.05 kg were used. The beagles were housed in stainless steel cages and maintained on a 12 h light–dark cycle with an ambient temperature of 22 $\pm$ 1 ^∘^C. All animal experimental protocols were conducted in accordance with the Animal Protection Act and the guideline for the Care and Use of Laboratory animals in the Republic of Korea (No. 13023, dated January 20, 2015). The ethical approval was obtained from the Institutional Animal Care and Use Committee (IACUC) of the Daegu-Gyeongbuk Medical Innovation Foundation (No. KMEDI-23050401). All authors complied with Animal Research: Reporting of In Vivo Experiments (ARRIVE) guidelines.

### Experiment procedures and designation

After an overnight fast (more than 12 h), the beagles were pre-anesthetized intramuscularly with 0.05 mg/kg of atropine and 2 mg/kg of xylazine. An intravenous line was placed using the aseptic technique into the dorsal vein of the hindfoot, and 2–3 mg/kg of alfaxalone was administrated. When each beagle reached an adequate state of anesthetic, it was intubated with an adequately sized endotracheal tube. The beagle was then placed on the table and connected to the anesthetic machine and ventilator (Supplementary Fig. 1). General anesthesia was maintained with inhaled 1–2% isoflurane delivered through a precision vaporizer. INV-001 was injected intradermally into four dorsal webspace at each hind limb using a small syringe (Supplementary Fig. 2). The injection sites of each dorsal webspace had to be massaged for approximately 2 min after INV-001 administration to facilitate absorption in the lymphatic vessels.

**Figure 2:**
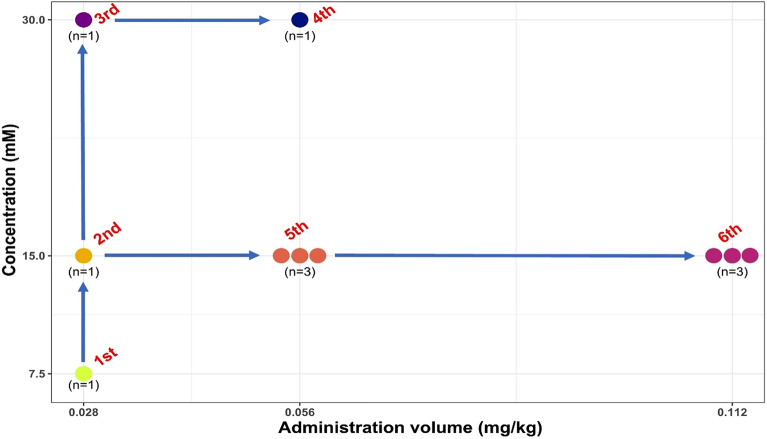
The schematic depicts the adaptive optimal dose finding study design.

First, we performed a dose-finding study using variable concentrations (7.5, 15, and 30 mM) and administration volumes (0.028, 0.056, and 0.112 mg Fe/kg) to determine the optimal concentration and injection volume of INV-001 for MRL in healthy beagles. Based on the qualitative visualization score analysis of lymphatic vessels and lymph nodes, we increased the administration concentrations and volumes of INV-001 from 7.5 mM and 0.028 mg Fe/kg (1st) to 15 mM and 0.012 mg Fe/kg (6th), respectively, according to the adaptive optimal dose-finding study design (Fig. 2). The detailed experimental design is summarized in Table 1. In the next phase, the reproducibility studies for determined optimal volume (0.067 and 0.133 mL/kg which correspond to 0.056 and 0.112 mg Fe/kg, respectively) with a concentration of 15 mM were performed. Lastly, an excretion study of INV-001 was conducted in three healthy beagles. The excretion study was performed to ascertain the presence of INV-001 at various concentrations and administration volumes (15 mM with 0.056 mg Fe/kg, 30 mM with 0.028 mg Fe/kg, and 30 mM with 0.056 mg Fe/kg) in the body within 48 h after administration, utilizing T_1_, T_2_, and T_2_* relaxation time measurements.

**Table 1:** Adaptive optimal dose finding study design (qualitative and quantitative analysis).

	No. of beagles
(1) Dose finding study for concentration and volume escalation	
1. Concentration (7.5 mM) and volume (0.028 mg Fe/kg)	1
2. Concentration (15 mM) and volume (0.028 mg Fe/kg)	1
3. Concentration (30 mM) and volume (0.028 mg Fe/kg)	1
4. Concentration (30 mM) and volume (0.056 mg Fe/kg)	1
5. Concentration (15 mM) and volume (0.056 mg Fe/kg)	1
6. Concentration (15 mM) and volume (0.112 mg Fe/kg)	1
(2) Reproducibility study for determined volume (with conc. of 15 mM)	
1. Concentration (15 mM) and volume (0.056 mg Fe/kg)	3
2. Concentration (15 mM) and volume (0.112 mg Fe/kg)	3
(3) Excretion study (48-h delay)	3
Total	15

### MRL acquisition and scan parameters

All of the MRL images were taken with the healthy beagle in the supine position using a 3.0-Tesla (T) whole body clinical magnetic resonance imaging scanner (MAGNETOM Skyra, Siemens Healthcare, Erlangen, Germany) with a maximum gradient strength of 45 mT/m and a slew rate of 200 T/m/s). The MRL images were obtained before and after contrast agent administration. We used two 18-channel body coils (Siemens Healthcare) with 18 integrated preamplifiers to image a wide range of the lower lymphatic vessels from the pelvis to the toes. In addition, the dual echo three-dimensional (3D) volumetric interpolated breath-hold examination (VIBE) sequence with fat suppression (Dixon technique) was used to acquire T_1_ weighted imaging in the coronal orientation with two stacks. The utilized scan parameters were as follows: field of view = 300 $\times$ 300 $\times$ 115 mm, repetition time (TR) = 6.06 ms, echo time (TE) = 2.46 / 3.69 ms, voxel size = 0.8 $\times$ 0.8 $\times$ 0.8 mm, acquisition matrix = 384 $\times$ 307, acceleration factor = 2, flip angle = 20^∘^, average = 1, and slice thickness/slice gap = 0.8/0 mm. A more detailed summary of the imaging parameters used for MRL is shown in Table 2. After contrast agent administration, MRL imaging was performed in five cycles (30, 40, 50, 60, and 70 min) on a coronal 3D VIBE sequence with fat suppression, except for the administration of 7.5 mM and 0.028 mg Fe/kg. In the case of the administration of 7.5 mM and 0.028 mg Fe/kg, there was no visualization of lymphatic vessels except for the popliteal lymph node. Thus, MRL images were no longer acquired after 50 min. Furthermore, variable TR, turbo spin echo (TSE) with multi-echo, and gradient with multi-echo sequence were used to acquire T_1_, T_2_, and T_2_* relaxation times, respectively. The utilized scan parameters are shown in Supplementary Table 1.

**Table 2:** A detailed summary of the scan parameters for magnetic resonance (MR) lymphangiography.

3D T_1_ VIBE sequence	
TR (ms)	6.06
TE (ms)	2.46 / 3.69
^a^Acceleration factor	2
Phase encoding direction	Right to Left
Phase oversampling	30%
Flip angle	20 ^∘^
Bandwidth (Hz/pixel)	690
FOV (mm)	300 $\times$ 300
Acquisition matrix	384 $\times$ 307
Number of slices	144
Scan orientation	Coronal plane
Slice thickness / gap (mm)	0.8 / 0
Fat suppression	Dixon technique

^a^Parallel imaging technique, known as generalized auto-calibrating partial parallel acquisition (GRAPPA), with acceleration factor 2 was used. MR lymphangiography was acquired with two stacks to visualize a wide range of lower lymphatic vessels. VIBE: volumetric interpolated breath-hold examination, TR: repetition time, TE: echo time, GRE: gradient echo, FOV: field of view.

### Quantitative analysis of image quality

**Figure 3:**
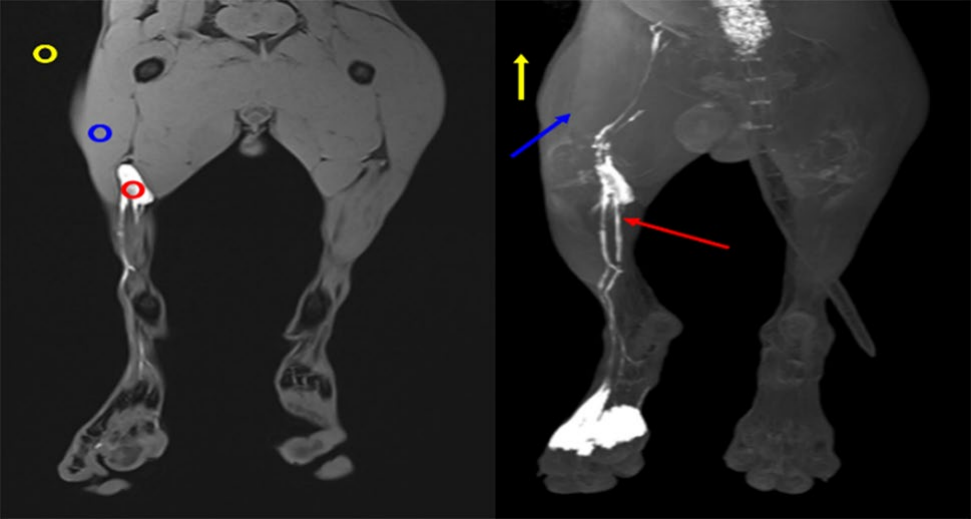
The region of interests (ROIs) to calculate signal-to-noise ratio and contrast-to-noise ratio. Red circle: popliteal lymph node, blue circle: muscle, yellow circle: background, red arrow: lymphatic vessel, blue arrow: muscle, yellow arrow: background.

The quantitative assessment for the degree of contrast enhancement in both lymph nodes and lymphatic vessels were performed using the signal-to-noise ratio (SNR) and contrast-to-noise ratio (CNR). The background region of interest (ROI) was drawn at an area where there were no anatomical structures in the beagles (Fig. 3). The SNR and CNR values were calculated using Image J (Bethesda, MD, USA; http://rsbweb.nih.gov/ij/) with the following equation by one reader:

$$\begin{gathered} {\text{SNR}}_{{{\text{LN}}}} {\text{ = SI}}_{{{\text{LN}}}} {\text{/ Mean SD}}_{{{\text{background}}}} \hfill \\ {\text{CNR}}_{{{\text{LN}}}} {\text{ = (SI}}_{{{\text{LN}}}} - {\text{ SI}}_{{{\text{muscle}}}} {\text{) / Mean SD}}_{{{\text{background}}}} \hfill \\ {\text{SNR}}_{{{\text{LV}}}} {\text{ = SI}}_{{{\text{LV}}}} {\text{/ Mean SD}}_{{{\text{background}}}} \hfill \\ {\text{CNR}}_{{{\text{LV}}}} {\text{ = (SI}}_{{{\text{LV}}}} - {\text{SI}}_{{{\text{muscle}}}} {\text{) / Mean SD}}_{{{\text{background}}}} \hfill \\ \end{gathered}$$

where SI_LN_, SI_LV,_ SI_muscle_, and SD_background_ indicate the signal intensities of the popliteal lymph node, lymphatic vessels, muscles, and standard deviation of background, respectively. In addition, the relaxation time of T_1_, T_2_, and T_2_* in the acquired map images were calculated using MATLAB (R2016b; MathWorks, Natick, MA, USA).

### Qualitative analysis of image quality

An image quality assessment with regards to the visualization of both lymph nodes and lymphatic vessels was performed independently by two radiologists to identify the optimal concentration and administration volume using a visual grading analysis based on the criteria of qualitative scores. The criteria used were 1) venous contamination, 2) dermal backflow, 3) lymph node congestion, and 4) score for the visualization of lymph nodes and lymphatic vessels. A detailed summary of the criteria for qualitative scores is presented in Table 3.

**Table 3:** Criteria for qualitative score.

Description	Qualitative score			
	0	1	2	3
Venous contamination	Present	Absent	–	–
Dermal backflow	Present	Absent	–	–
Lymph node congestion	Present	Absent	–	–
Visualization	Poor	Moderate	Good	Excellent

We used this scoring system (where 1 denotes absence) to ensure that a higher score reflected better image quality.

### Statistical analysis

The interobserver agreements for qualitative assessment were evaluated using weighted kappa values. The strength of the interobserver agreement according to the weighted kappa value was assessed as follows: ≤ 0.2, poor; 0.21–0.40, fair; 0.41–0.60, moderate; 0.61–0.80, good; and 0.81–1.00, excellent^18,19^. The Kolmogorov–Smirnov test was utilized toverify that the quantitative analysis and relaxation times conformed to a normal distribution. Subsequently, depending on the results of the Kolmogorov–Smirnov test, either the Kruskal–Wallis test or analysis of variance (ANOVA) and either the Wilcoxon signed-rank test or paired t-test were employed to compare the quantitative analyses in both the dose finding and reproducibility studies. The values of T_1_, T_2_, and T_2_* relaxation time as a function of anatomical lesions were compared using a paired Student’s *t*-test. Statistical analyses were performed using IBM SPSS Statistics for Windows/Macintosh, v. 26.0 (IBM Corp., Armonk, NY, USA). For all of the statistical analyses, a two-sided level of *p* < 0.05 was considered to indicate a statistically significant difference.

## Results

### Characterization of INV-001

We analyzed the colloidal properties of INV-001 using DLS. The measured hydrodynamic size and zeta-potential were 3.6 nm (Fig. 1b) and − 2.95 mV, respectively. In the colloidal stability test, INV-001 was stably dispersed without aggregation in various pH ranges of 5 to 9 and NaCl concentrations up to 1 M for 7 days (Supplementary Fig. 3). In the MRI phantom study, INV-001 exhibited a bright signal in tested concentrations in T_1_-weighted images acquired using a 3 T MRI scanner (Fig. 1C). The measured *r*_1_ and *r*_2_ values were 3.8 and 4.6 mM^−1^ s^−1^, respectively (Fig. 1D,E). The *r*_2_/*r*_1_ ratio, a crucial parameter for evaluating the performances of T_1_ MRI contrast agents, was 1.2. Considering the optimal T_1_ contrast effects with an ideally low *r*_2_/*r*_1_ value of ~ 1, INV-001 exhibited excellent T_1_ contrast effects. For the toxicity test of INV-001, we conducted a 4 week repeated intradermal dose toxicity study in beagles. The injection doses were 1.56, 3.12, and 6.24 mg Fe/kg (0.028, 0.056, 0.112 mmol Fe/kg), which corresponded to 14-, 28-, and 56-fold excesses of the maximum dosage (0.112 mg Fe/kg or 0.002 mmol Fe/kg) utilized in MRL study. Throughout the 4 weeks observation period following intradermal injection, no deaths were observed in any of the tested animals. Additionally, no significant differences in the body weight patterns were observed between the control group and the INV-001-injected groups (Supplementary Fig. 4). These results indicate the outstanding T_1_ MRI contrast effect and biocompatibility of INV-001.

### Dose finding study

**Figure 4:**
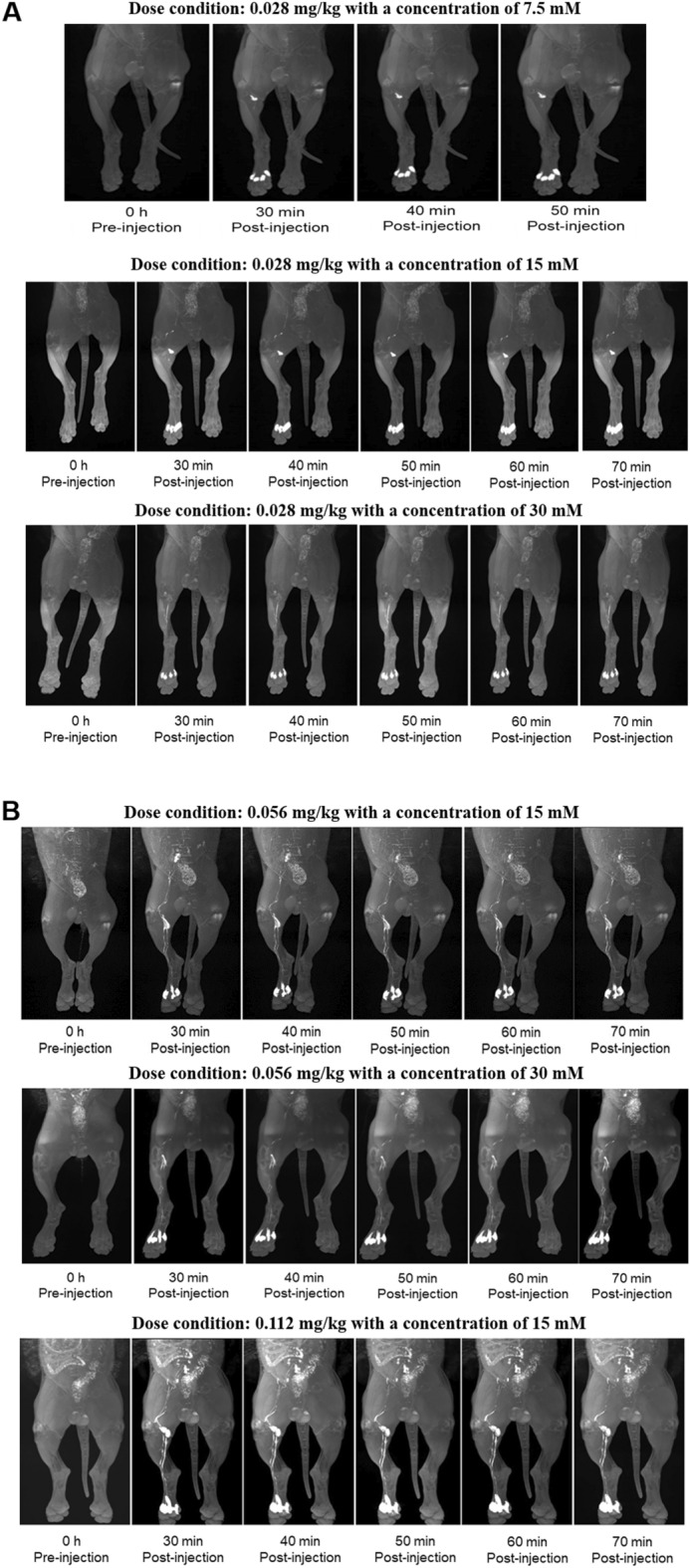
The magnetic resonance lymphangiography images as a function of administration time, volume, and concentrations. (**A**) the variable concentration groups (7.5, 15, and 30 mM) with a fixed administration of 0.028 mg Fe/kg, (**B**) at a dose of 0.056 mg Fe/kg with concentrations of 15 mM and 30 mM.

The results of the qualitative score analysis of lymphatic vessels for the adaptive optimal dose-finding study at various dosing conditions of 0.028, 0.056, and 0.112 mg Fe/kg with different concentrations (7.5, 15, and 30 mM) are demonstrated in Table 4. In all tested conditions, venous contamination was not observed. The qualitative image analysis demonstrated that the doses of 0.028 mg Fe/kg with concentrations of 7.5, 15, and 30 mM showed a relatively low total score of 2.5, 4.5, and 3.5, respectively (Fig. 4A and Table 4). At a dose of 0.056 mg Fe/kg with concentrations of 15 and 30 mM, the total scores were 6 and 5, respectively. Since the higher total score was achieved at the concentration of 15 mM rather than 30 mM at same dose of 0.056 mg Fe/kg, a further higher dose of 0.112 mg Fe/kg at a concentration of 15 mM was tested, and a total score of 6 was achieved (Fig. 4B and Table 4). There was moderate to excellent interobserver agreement between the two radiologists regarding the visual grading for qualitative assessment in dose finding study (weighted kappa, κ = 0.51–1).

**Table 4:** The results of qualitative scoring at variable dose conditions of INV-001.

Order	Group	Beagle ID	Readers	Qualitative scoring				
				Venous contamination	Dermal backflow	Lymph node congestion	Visualization	Total
1	INV-001 7.5 mM 0.028 mg Fe/kg	#01	1	1.0	1.0	0.0	0.0	2.0
			2	1.0	1.0	1.0	0.0	3.0
		Average		1.0	1.0	0.5	0.0	2.5
2	INV-001 15 mM 0.028 mg Fe/kg	#01	1	1.0	1.0	1.0	2.0	5.0
			2	1.0	1.0	1.0	1.0	4.0
		Average		1.0	1.0	1.0	1.5	4.5
3	INV-001 30 mM 0.028 mg Fe/kg	#02	1	1.0	1.0	0.0	1.0	3.0
			2	1.0	1.0	1.0	1.0	4.0
		Average		1.0	1.0	1.0	1.0	3.5
4	INV-001 30 mM 0.056 mg Fe/kg	#03	1	1.0	1.0	1.0	2.0	5.0
			2	1.0	1.0	1.0	2.0	5.0
		Average		1.0	1.0	1.0	2.0	5.0
5	INV-001 15 mM 0.056 mg Fe/kg	#02	1	1.0	1.0	1.0	3.0	6.0
			2	1.0	1.0	1.0	3.0	6.0
		Average		1.0	1.0	1.0	3.0	6.0
6	INV-001 15 mM 0.112 mg Fe/kg	#02	1	1.0	1.0	1.0	3.0	6.0
			2	1.0	1.0	1.0	3.0	6.0
		Average		1.0	1.0	1.0	3.0	6.0

**Figure 5:**
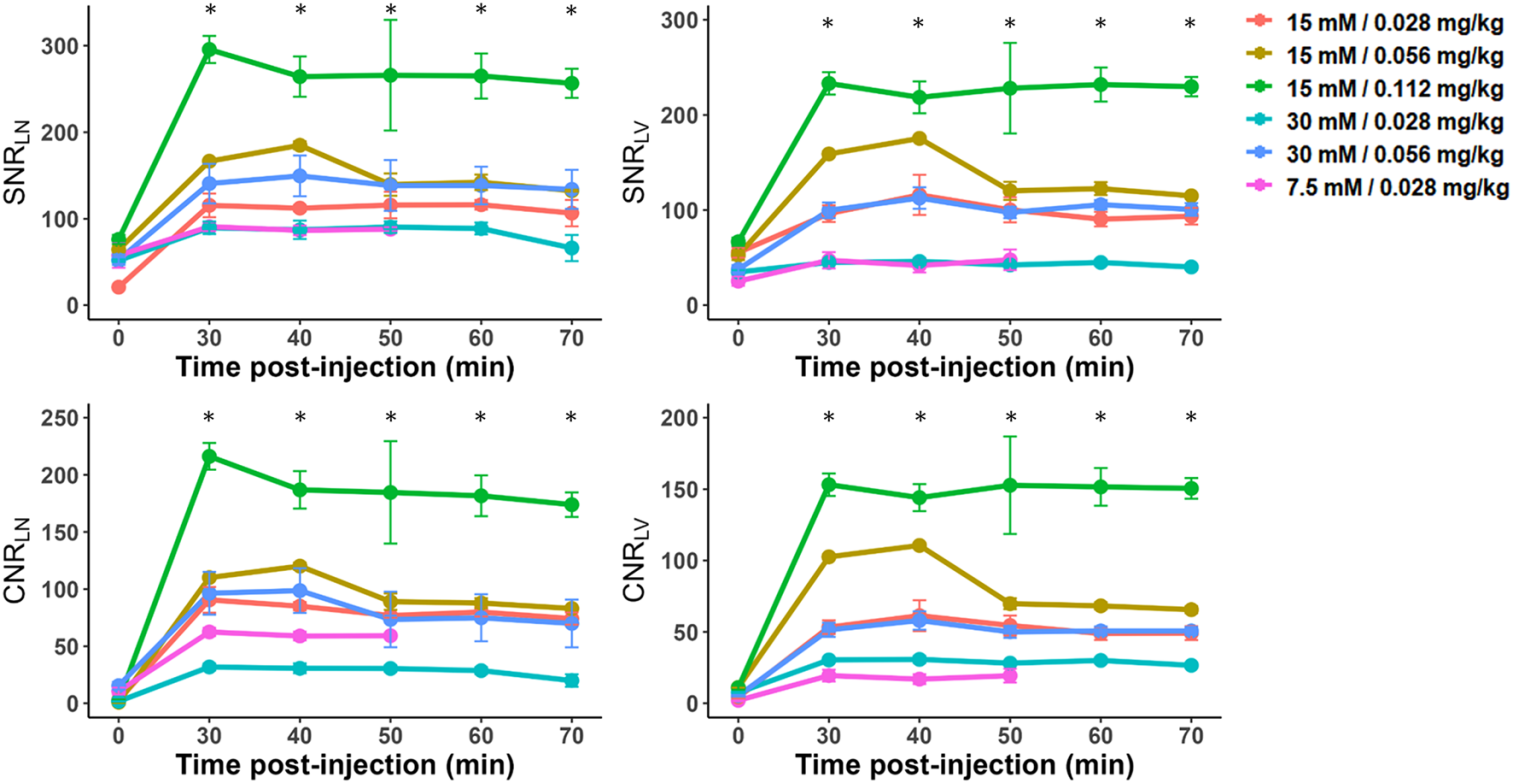
Time-dependent signal-to-noise ratio (SNR)_LN_, SNR_LV_, contrast-to-noise ratio (CNR)_LN_, and CNR_LV_ values at different dose conditions for the adaptive optimal dose finding study. SNR _LN,_ SNR_LV,_ CNR_LN,_ and CNR_LV_ values are mean $\pm$ standard error. LN: lymph node, LV: lymphatic vessel. The statistical significances are indicated with an asterisk (*), representing *p* < 0.05. Areas without an asterisk denote results that are not statistically significant, with *p* > 0.05.

In the quantitative analysis, a dose condition of 0.056 and 0.112 mg Fe/kg at a concentration of 15 mM showed the best contrast enhancement of lymphatic vessels and nodes. Specifically, SNR_LN_, SNR_LV,_ CNR_LN_, and CNR_LV_ showed the highest values of 295.62 $\pm$ 27.08, 233.14 $\pm$ 20.31, 216.22 $\pm$ 20.21, and 153.16 $\pm$ 13.61, respectively, at a dose of 0.112 mg Fe/kg with a concentration of 15 mM (Fig. 5). Based on these quantitative and qualitative analyses, the dose condition of 0.056 and 0.112 mg Fe/kg with a concentration of 15 mM were selected for the dose expansion study for the reproducibility assessment.

### Reproducibility study

**Figure 6:**
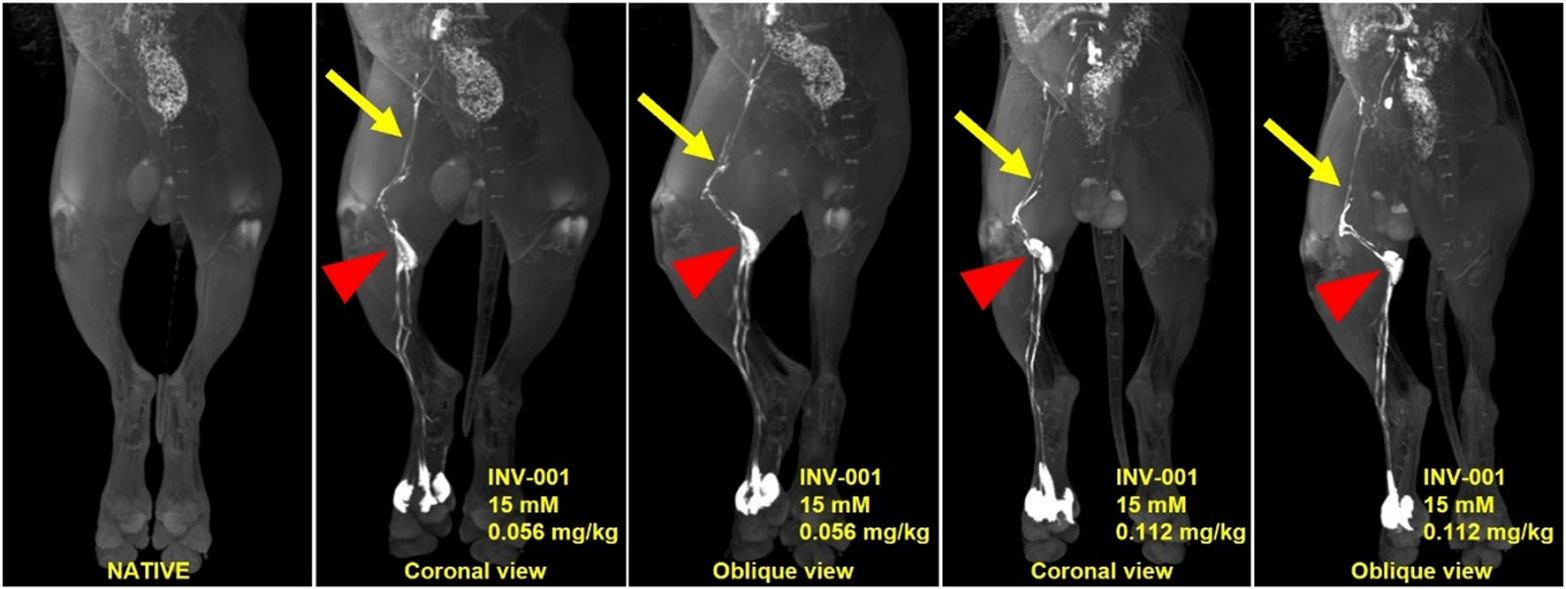
Reproducibility study images showing a good visualization for both lymphatic vessels and nodes without venous contamination and dermal backflow.

The results of the dose expansion study for the reproducibility evaluation are presented in Table 5. All acquired MRL imaging clearly demonstrated the visibility of lymphatic vessels and lymph nodes without venous contamination. In the qualitative analysis, the doses of 0.056 and 0.112 mg Fe/kg with a concentration of 15 mM received total scores of 5.33 and 5.66, respectively (Table 5). There was good to excellent interobserver agreement between the two radiologists regarding the visual grading for qualitative assessment in reproducibility study (weighted kappa, κ = 0.76–1). Overall, the qualitative analysis in both groups showed a good visualization for both lymphatic vessels and lymph nodes. No venous contamination and dermal backflow were observed for all tested conditions (Fig. 6).

**Table 5:** The results of qualitative scoring at doses of 0.056 and 0.112 mg Fe/kg with a concentration of 15 mM.

Group	Beagle ID	Readers	Qualitative scoring				
			Venous contamination	Dermal backflow	Lymph node congestion	Visualization	Total
INV-001 15 mM 0.056 mg Fe/kg	#01	1	1.0	1.0	0.0	1.0	3.0
		2	1.0	1.0	1.0	2.0	5.0
	#02	1	1.0	1.0	1.0	3.0	6.0
		2	1.0	1.0	1.0	3.0	6.0
	#03	1	1.0	1.0	1.0	3.0	6.0
		2	1.0	1.0	1.0	3.0	6.0
	Average		1.0	1.0	1.0	2.5	5.33
INV-001 15 mM 0.112 mg Fe/kg	#01	1	1.0	1.0	1.0	2.0	5.0
		2	1.0	1.0	1.0	2.0	5.0
	#02	1	1.0	1.0	1.0	3.0	6.0
		2	1.0	1.0	1.0	3.0	6.0
	#03	1	1.0	1.0	1.0	3.0	6.0
		2	1.0	1.0	1.0	3.0	6.0
	Average		1.0	1.0	1.0	2.66	5.66

**Figure 7:**
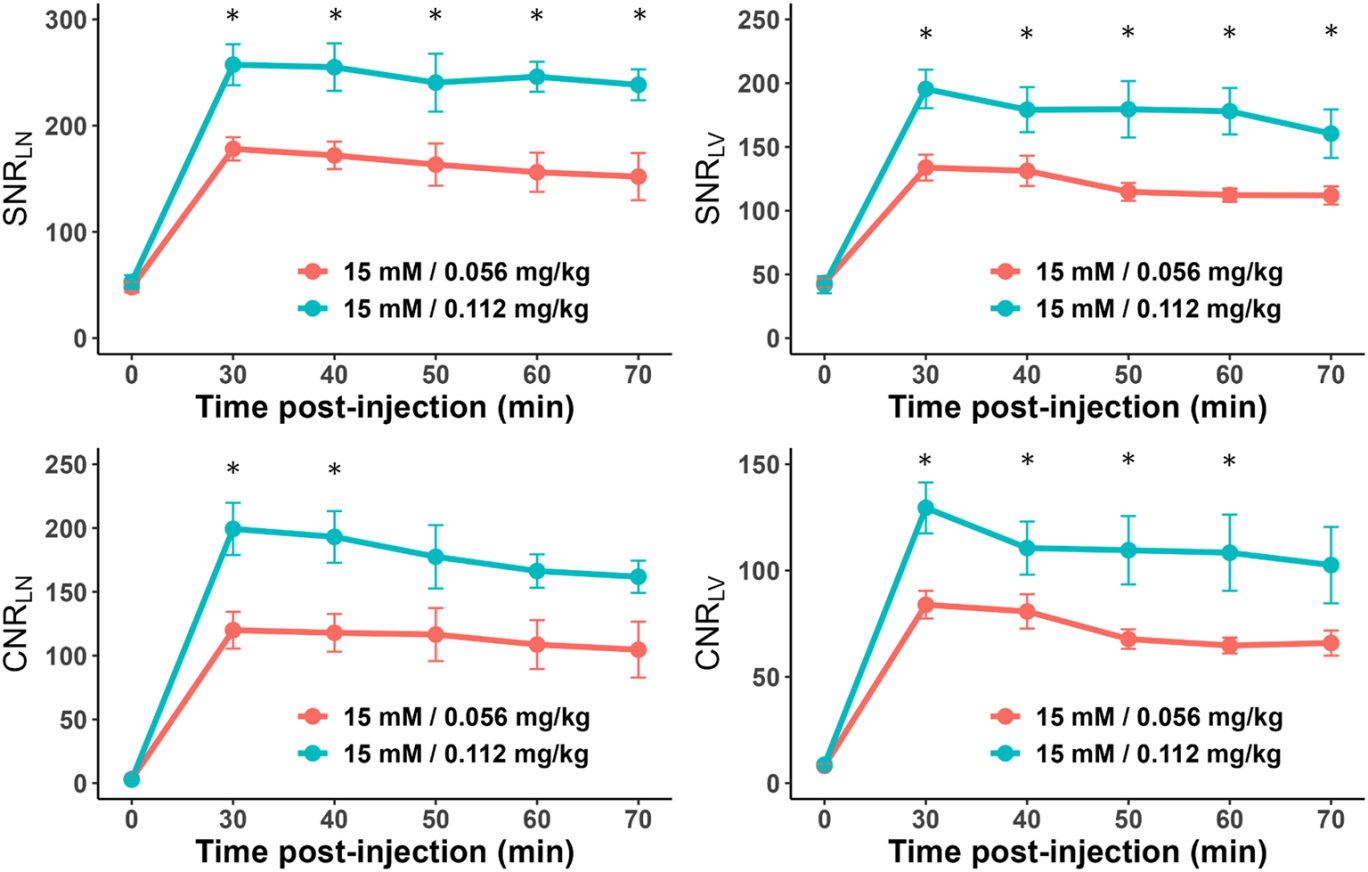
Time-dependent signal-to-noise ratio (SNR)_LN_, SNR_LV_, contrast-to-noise ratio (CNR)_LN_, and CNR_LV_ values at doses of 0.056 and 0.112 mg Fe/kg with a concentration of 15 mM for the reproducibility study. SNR _LN,_ SNR_LV,_ CNR_LN,_ and CNR_LV_ values are mean $\pm$ standard error. LN: lymph node, LV: lymphatic vessel. The statistical significances are indicated with an asterisk (*), representing *p* < 0.05. Areas without an asterisk denote results that are not statistically significant, with *p* > 0.05.

In the quantitative analysis, SNR_LN_, SNR_LV,_ CNR_LN_, and CNR_LV_ exhibited the maximum values of 178.12 $\pm$ 32.84, 133.76 $\pm$ 30.33, 119.96 $\pm$ 43.40, and 83.94 $\pm$ 19.43 for the dose group of 0.056 mg Fe/kg and 257.34 $\pm$ 61.10, 195.44 $\pm$ 45.26, 199.37 $\pm$ 61.58, and 138.32 $\pm$ 48.03 for the dose group of 0.112 mg Fe/kg, respectively, at 30 min after the administration of INV-001 (Fig. 7). Furthermore, these values exhibited a gradual decrease until the 70-min mark, with a range of decrease rates between 7.3 and 22.8%, resulting in a continuous visualization of the lymphatic vessels and lymph nodes. Compared to the dose group of 0.056 mg Fe/kg, all tested results for the dose group of 0.112 mg Fe/kg showed a higher value in both lymphatic vessels and lymph nodes. These results indicate that INV-001 effectively enhanced lymphatic vessels and lymph nodes in a dose-dependent manner. Based on both quantitative and qualitative analyses, an administration dose of 0.056 and 0.112 mg Fe/kg with a concentration of 15 mM demonstrated good reproducibility in visualizing lymphatic vessels and lymph nodes without venous contamination. Therefore, it can be determined that an administration dose higher than 0.056 mg Fe/kg with a concentration of 15 mM INV-001 is sufficient for MRL in beagle dogs. All quantitative results are presented in Supplementary Tables 2, 3, 4, and 5.

### Excretion study

To confirm the excretion of INV-001, T_1_, T_2_, and T_2_* relaxation time of the liver and kidneys were analyzed at 0 (pre-injection) and 48 h after administration. INV-001 was administered at a dose of 0.056 mg Fe/kg with a concentration of 15 mM. Compared to pre-injection (0 h), there were no statistically significant differences after 48 h for T_1_, T_2_, and T_2_* relaxation time measured at both the liver and kidneys (*p* > 0.05), except for T_2_* relaxation time of the liver (*p* < 0.05) as shown in Supplementary Fig. 5 and Table 6. Additionally, the INV-001 did not remain in the injection site. Therefore, it is considered that the INV-001 was effectively excreted from the body within 48 h.

## Discussion

In this study, we clearly demonstrated contrast enhancement without venous contamination, providing that only lymph nodes and lymphatic vessels were selectively imaged using the optimal administration concentration and volume for MRL with INV-001 in healthy beagles.

While GBCA-based approaches are hindered by venous contamination, the hydrodynamic size of INV-001 (3.6 nm) is smaller than the renal clearance threshold. Consequently, following intradermal administration, it does not enter the venous circulation but is exclusively cleared through the lymphatic system and ultimately excreted in the urine. This pathway ensures that INV-001 remains confined to the lymphatic vessels, thereby preventing venous contamination.

The findings based on quantitative and qualitative analyses revealed that the optimal concentration was 15 mM and the recommended administration volumes were 0.067 and 0.133 mL/kg, corresponding to dosages of 0.056 and 0.112 mg Fe/kg for MRL in beagles. Although the typical concentrations of gadolinium-based MR contrast agents used in clinical practice for MRL range from 500 to 1000 mmol/L with a recommended volume of 0.1–0.2 mL/kg^8,20,21^, our research achieved significant clinical benefits by visualizing only the lymphatic vessels with a concentration of as low as 15 mmol/L with 0.067 and 0.13 mL/kg and without venous contamination. These results hold significant implications for the academic community and contribute to our understanding of the appropriate administration parameters for MRL with INV-001 in preclinical studies involving beagle dogs.

As the efforts of reducing venous contamination are crucial in the MRL field, some studies demonstrated that the dual-agent relaxation contrast technique using ferumoxytol and GBCAs result in the suppression of the contamination of the venous signal, revealing an isolated lymphatic signal^15,22^. However, ferumoxytol, which was developed as a treatment drug for anemia, induces a serious hypersensitivity and anaphylactoid-type reaction, so the use of ferumoxtran for imaging indication is not approved by the Food and Drug Administration (FDA)^23^. In addition, a study showed that the peak enhancement and optimal delineation of lymphatic vessels were observed between 35 and 45 min after the administration of INV-001^24^. The findings were similar to those in our study; however, due to differences in MR scan parameters and experimental subjects that can influence image quality, absolute significance cannot be attributed to the results.

Our study had some limitations. First, the excretion study of INV-001 was performed without the breath-hold technique. The image quality of anatomical lesions, such as those of the liver, are known to be influenced by breath-holding. Contrary to other relaxation times, there was a statistically significant difference in the T2* relaxation time of the liver before and after the administration of the contrast agent. This difference might due to uncontrolled free breathing and the MR sequence used during the image acquisition. Unlike other sequences used for measuring relaxation time, T2* relaxation time sequence was performed using a gradient echo that was basically made without multiple refocusing pulses (180^∘^) rephasing the phase shift resulting from magnetic field inhomogeneities. A possible explanation is that the absence of multiple refocusing pulses have various effects on blurred image quality, including susceptibility and inhomogeneities, and more variation of measured relaxation time. Secondly, relaxation times were measured only at 48 h post-administration of INV-001 due to constraints at our experimental facility, not at 24 h as initially intended. Although no significant changes were detected at 48 h and MIP images indicated no remaining contrast agent, the lack of data from earlier time points limits our ability to confirm if transient changes occurred and subsequently normalized. Future studies should expand time point assessments to provide a more comprehensive evaluation. Lastly, we do not perform control group experiments using the GBCAs to compare our study results. However, it is well-known from various clinical studies that GBCAs, being water-soluble and diffusible, are limited in visualizing both lymphatic vessels and venous contamination in MRL^2,13,14^. To clearly show the clinical effect of INV-001, further efforts in large-scale clinical trials to validate the effectiveness of INV-001 in visualizing lymphatic vessels and lymph nodes without venous contamination in MRL are necessary. Despite these limitations, the efficient visualization of only the lymphatic vessels and lymph nodes without venous contamination in our research holds significant value. Moreover, the results presented in our study are of considerable importance as they provide fundamental information for future clinical trials aiming to visualize only the lymphatic vessels in MRL imaging. This valuable insight is expected to contribute significantly to such trials.

In MRL, INV-001, a new type of contrast agent consisting of dextran core-iron oxide shell, demonstrated contrast-enhanced visualization of lymphatic vessels and nodes without venous contamination. The optimal administration concentration and volume for MRL with INV-001 was found to be 15 mM with 0.056 and 0.112 mg Fe/kg, respectively, in healthy beagles.

### Supplementary Information

Supplementary Information.

## Data Availability

All of the data are available from the corresponding author upon reasonable request.
