# Supplementary material for: Analysis on efficacy of magnetic resonance lymphangiography using INV-001 in healthy beagle dogs
Source: Sci Rep. 2024 May 7;14:10502. doi: 10.1038/s41598-024-61104-4 (PMC11076550; doi:10.1038/s41598-024-61104-4)
Supplement: Supplementary file 1 — Supplementary Information. [file 41598_2024_61104_MOESM1_ESM.docx]

**Supplementary Figure 1.** Images showing Beagle dogs' position for MR scanning and anesthesia setup

**
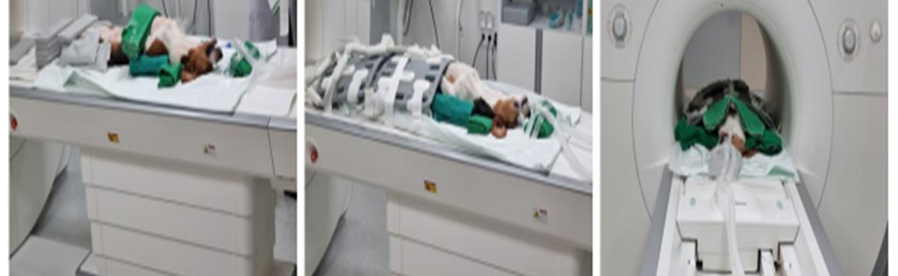
**

**Supplementary Figure 2.** Injection site in Beagle dogs for MRL

**
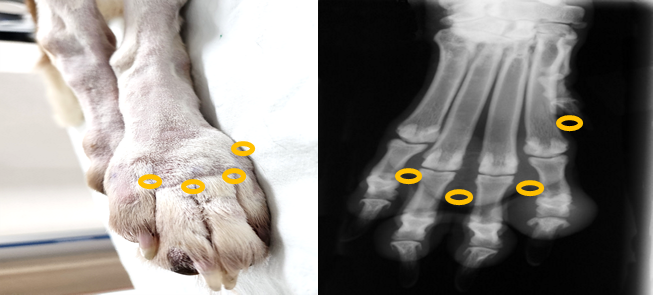
**

**Supplementary Figure 3.** Colloidal stability of INV-001.

Hydrodynamic size of INV-001 in pH 5,7, and 9 buffer solution (a) and 250, 500, and 1000 mM NaCl solution (b).

**
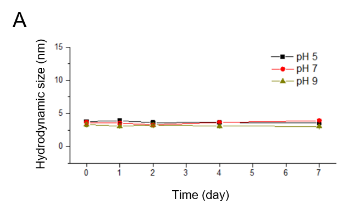

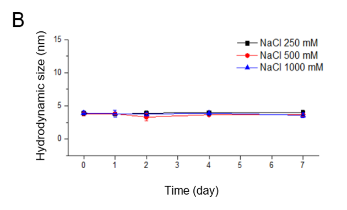
**

**Supplementary Figure 4.** Dog body weight test after the intradermal injection of INV-001 (injection dose = 1.56, 3.12, and 6.24 mg Fe/kg, n=3 male beagle dog for each group).

**
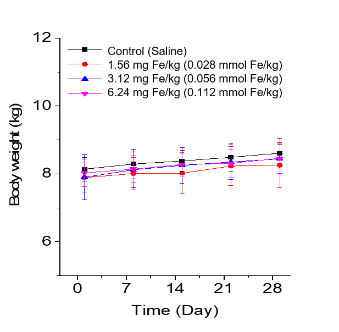
**

**Supplementary Figure 5**. T_1_ (A), T_2_ (B), and T_2_* (C) relaxation time obtained at the before (0 hour) and after (48 hours) administration of INV-001 analyzed at the liver and kidneys

**
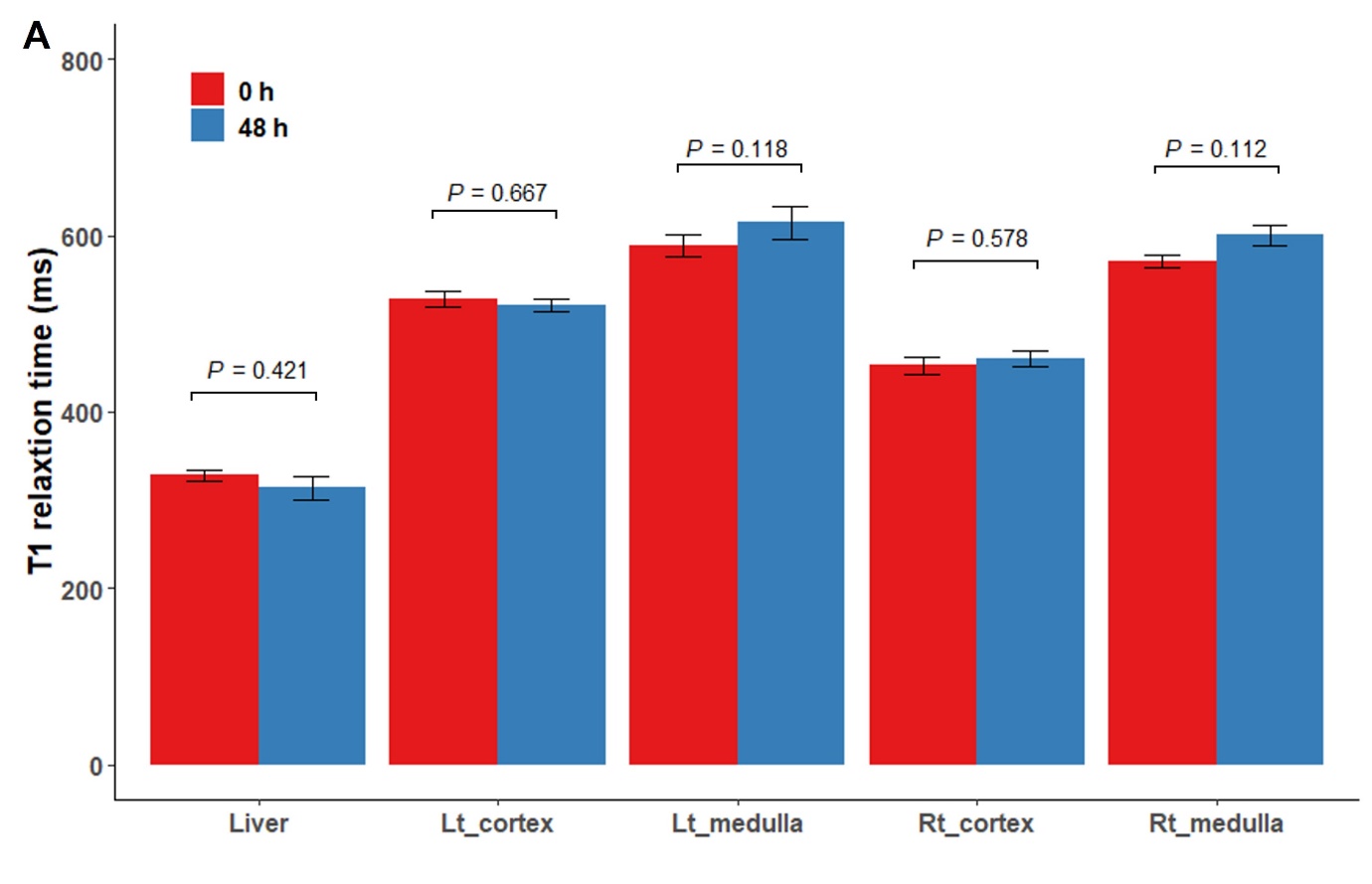
**

**
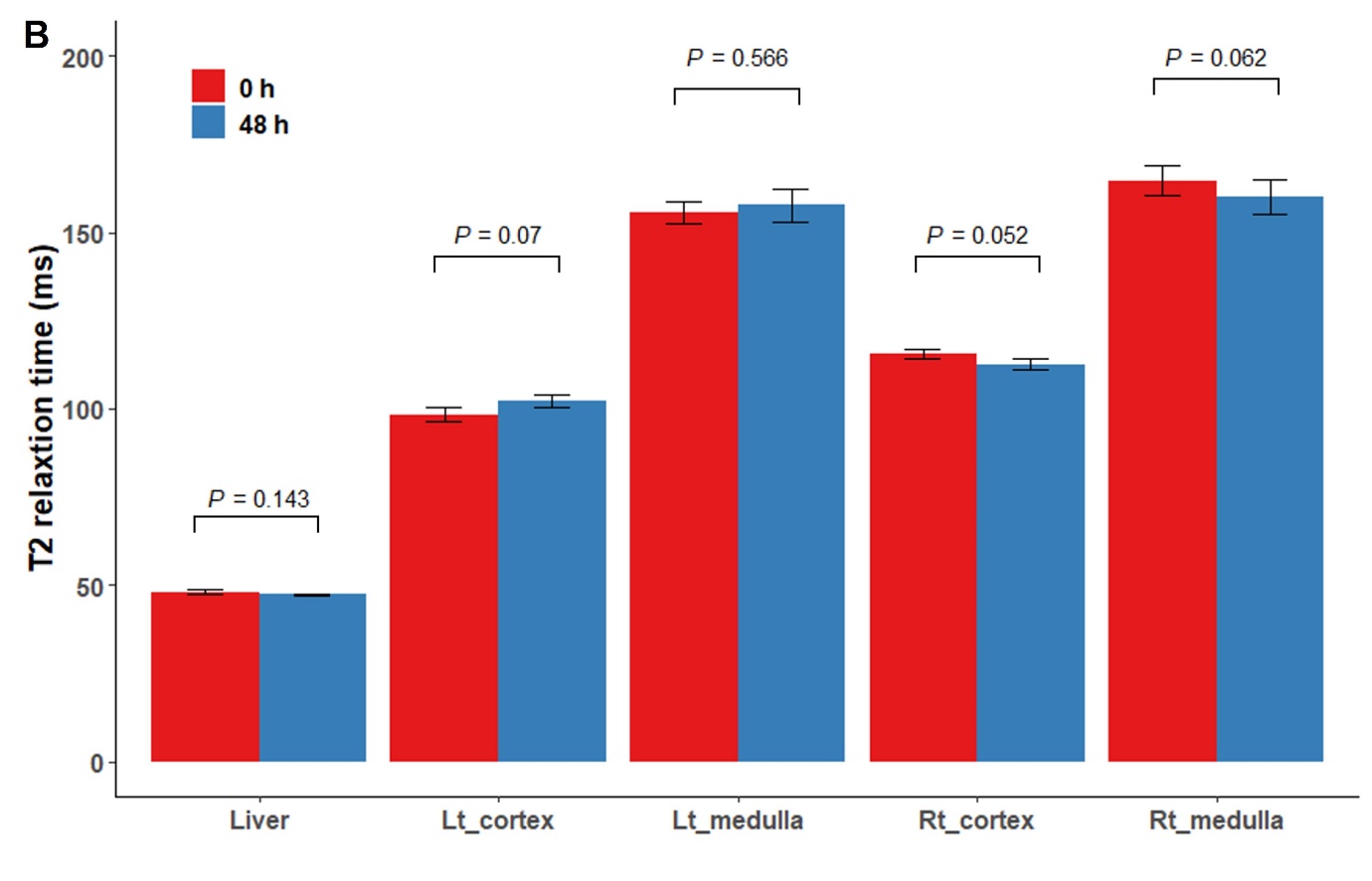
**

**
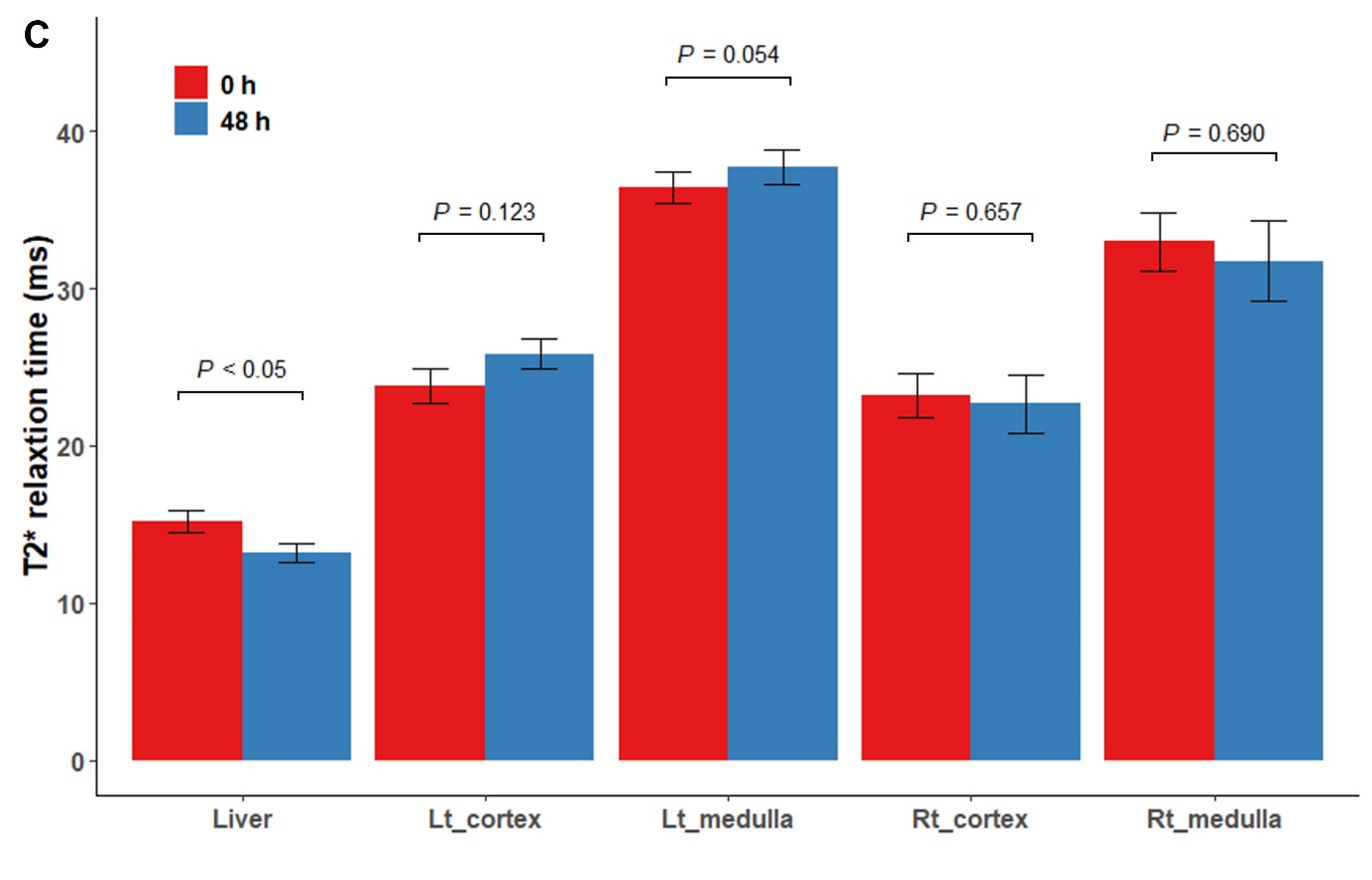
**

**Supplementary Figure 6. Excretion images of INV-001 injection site in Beagle Dog**

**
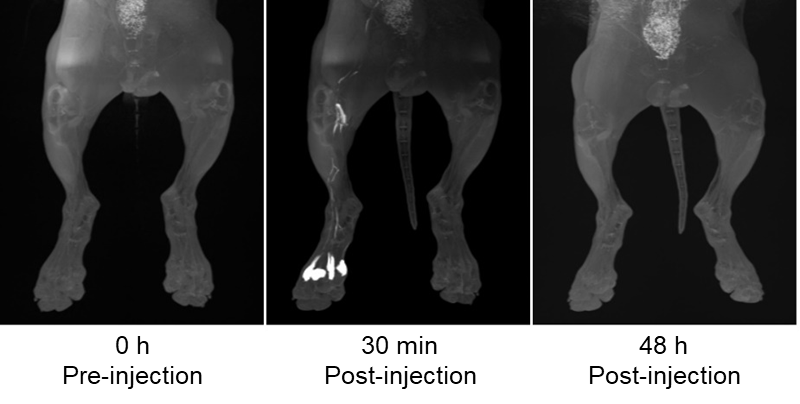
**

**Supplementary Table 1**. A detailed summary of the scan parameters for calculating relaxation time

|  | **T_1_ relaxation time** | **T_2_ relaxation time** | **T_2_* relaxation time** |
| --- | --- | --- | --- |
| FOV | 300 $\times$ 244 | 300 $\times$ 244 | 300 $\times$ 244 |
| Slice thickness/gap (mm) | 7 / 0.7 | 7 / 0.7 | 7 / 0.7 |
| TR (ms) | 20 / 40 / 60 / 80 / 120 / 200 | 2400 | 300 |
| TE (ms) | 6.6 | 13.8 / 27.6 / 41.4 / 55.2 / 69 / 82.8 / 96.6 / 110.4 | 2.46 / 4.92 / 7.38 / 9.84 / 12.3 / 14.76 / 17.22 |
| Average | 1 | 1 | 1 |
| No. of slices | 20 | 15 | 20 |
| Scan orientation | Axial | Axial | Axial |
| Acquisition matrix | 256 $\times$ 187 | 320 $\times$ 208 | 256 $\times$ 166 |

Note: FOV: field of view, TR: repetition time, TE: echo time, T_1_, T_2_, and T_2_* relaxation time images were acquired using variable TR, turbo spin echo with multi-echo, and gradient with multi-echo sequence, respectively.

**Supplementary Table 2**. Individual SNR in the lymph node

|  | Beagle  ID | N | SNR_LN_ | | | | | | | | | |
| --- | --- | --- | --- | --- | --- | --- | --- | --- | --- | --- | --- | --- |
|  |  |  | 0 min | 30 min | | 40 min | | 50 min | | 60 min | | 70 min |
| †INV-001  7.5 mM  0.028 mg/kg | #01 | 1 | 46.82 | | 83.18 | | 94.01 | | 92.67 | | N/A | N/A |
|  |  | 2 | 85.66 | | 88.61 | | 90.16 | | 85.76 | | N/A | N/A |
|  |  | 3 | 40.12 | | 100.67 | | 75.70 | | 85.33 | | N/A | N/A |
|  | Mean | 57.53 | | | 90.82 | | 86.62 | | 87.92 | | N/A | N/A |
|  | SD | 24.58 | | | 8.95 | | 9.65 | | 4.11 | | N/A | N/A |
|  | SE | 14.19 | | | 5.16 | | 5.57 | | 2.37 | | N/A | N/A |
| INV-001  15 mM  0.028 mg/kg | #01 | 1 | 26.28 | | 92.67 | | 104.84 | | 146.41 | | 125.82 | 85.33 |
|  |  | 2 | 17.11 | | 113.68 | | 116.66 | | 99.97 | | 106.59 | 136.11 |
|  |  | 3 | 19.31 | | 140.11 | | 115.29 | | 101.01 | | 116.18 | 98.23 |
|  | Mean | 20.91 | | | 115.49 | | 112.26 | | 115.79 | | 116.21 | 106.56 |
|  | SD | 4.78 | | | 23.77 | | 6.46 | | 26.51 | | 9.61 | 26.39 |
|  | SE | 2.76 | | | 13.72 | | 3.73 | | 15.31 | | 5.55 | 15.23 |
| ‡INV-001  15 mM  0.056 mg/kg | #01 | 41.60 | | | 210.68 | | 203.80 | | 234.42 | | 222.31 | 228.91 |
|  | #02 | 64.40 | | | 166.42 | | 184.85 | | 139.49 | | 142.08 | 132.42 |
|  | #03 | 38.08 | | | 157.27 | | 127.33 | | 116.21 | | 104.16 | 94.86 |
|  | Mean | 48.03 | | | 178.12 | | 171.99 | | 163.37 | | 156.18 | 152.06 |
|  | S.D. | 14.62 | | | 32.84 | | 38.81 | | 59.63 | | 55.21 | 66.50 |
|  | SE | 4.87 | | | 10.94 | | 12.93 | | 19.87 | | 18.41 | 22.16 |
| ‡INV-001  15 mM  0.112 mg/kg | #01 | 36.52 | | | 294.55 | | 279.16 | | 273.19 | | 272.87 | 262.15 |
|  | #02 | 76.14 | | | 295.62 | | 264.31 | | 265.87 | | 265.01 | 256.58 |
|  | #03 | 46.11 | | | 181.84 | | 221.61 | | 182.51 | | 200.32 | 196.54 |
|  | Mean | 52.93 | | | 257.34 | | 255.02 | | 240.52 | | 246.06 | 238.42 |
|  | S.D. | 19.28 | | | 61.10 | | 70.64 | | 86.05 | | 44.62 | 45.97 |
|  | SE | 6.42 | | | 19.32 | | 22.34 | | 27.21 | | 14.11 | 14.53 |
| INV-001  30 mM  0.028 mg/kg | #02 | 1 | 54.50 | | 102.19 | | 108.34 | | 102.58 | | 92.96 | 96.49 |
|  |  | 2 | 50.20 | | 89.38 | | 75.06 | | 88.89 | | 98.06 | 52.92 |
|  |  | 3 | 50.28 | | 77.29 | | 78.38 | | 79.76 | | 75.31 | 49.23 |
|  | Mean | 51.66 | | | 89.62 | | 87.26 | | 90.41 | | 88.77 | 66.21 |
|  | SD | 2.45 | | | 12.45 | | 18.33 | | 11.48 | | 11.94 | 26.28 |
|  | SE | 1.42 | | | 7.19 | | 10.58 | | 6.63 | | 6.89 | 15.17 |
| INV-001  30 mM  0.056 mg/kg | #03 | 1 | 60.22 | | 95.88 | | 103.73 | | 130.27 | | 127.66 | 105.09 |
|  |  | 2 | 57.18 | | 155.19 | | 162.31 | | 92.19 | | 107.42 | 118.31 |
|  |  | 3 | 40.40 | | 170.88 | | 182.44 | | 192.76 | | 180.21 | 178.41 |
|  | Mean | 52.61 | | | 140.65 | | 149.49 | | 138.41 | | 138.43 | 133.93 |
|  | SD | 10.67 | | | 39.56 | | 40.88 | | 50.77 | | 37.57 | 39.07 |
|  | SE | 6.16 | | | 22.84 | | 23.60 | | 29.31 | | 21.69 | 22.55 |

Note: ^†^In case of administration dose of 0.028 mg/kg with a concentration of 7.5 mM, there was no visualization of lymphatic vessels except for the popliteal lymph node, and MRL images were not acquired after 50 minutes. **^‡^**Symbol indicates SNR was measured 3 times for each Beagle dog. SNR_LN_: signal-to-noise ratio­_lymph node_, SD: standard deviation, SE: standard error, N: number of measurements.

**Supplementary Table 3.** Individual CNR in the lymph node

|  | Beagle  ID | N | CNR_LN_ | | | | | | | | | |
| --- | --- | --- | --- | --- | --- | --- | --- | --- | --- | --- | --- | --- |
|  |  |  | 0 min | 30 min | | 40 min | | 50 min | | 60 min | | 70 min |
| †INV-001  7.5 mM  0.028 mg/kg | #01 | 1 | 8.06 | | 57.20 | | 64.38 | | 62.06 | | N/A | N/A |
|  |  | 2 | 16.63 | | 60.74 | | 60.93 | | 57.63 | | N/A | N/A |
|  |  | 3 | 7.11 | | 69.49 | | 51.51 | | 58.89 | | N/A | N/A |
|  | Mean | 10.61 | | | 62.47 | | 58.94 | | 59.20 | | N/A | N/A |
|  | SD | 5.24 | | | 6.32 | | 6.66 | | 2.48 | | N/A | N/A |
|  | SE | 3.02 | | | 3.65 | | 3.84 | | 1.43 | | N/A | N/A |
| INV-001  15 mM  0.028 mg/kg | #01 | 1 | 2.32 | | 72.26 | | 77.97 | | 75.03 | | 86.15 | 65.46 |
|  |  | 2 | 3.63 | | 111.15 | | 89.68 | | 80.55 | | 73.01 | 83.91 |
|  |  | 3 | 2.69 | | 88.35 | | 87.51 | | 74.94 | | 80.58 | 73.24 |
|  | Mean | 2.88 | | | 90.59 | | 85.05 | | 76.84 | | 79.91 | 74.20 |
|  | SD | 0.67 | | | 19.54 | | 6.23 | | 3.21 | | 6.59 | 9.25 |
|  | SE | 0.38 | | | 11.28 | | 3.59 | | 1.85 | | 3.81 | 5.34 |
| ‡INV-001  15 mM  0.056 mg/kg | #01 | 4.97 | | | 171.18 | | 165.18 | | 194.74 | | 181.31 | 184.21 |
|  | #02 | 0.91 | | | 110.06 | | 120.07 | | 89.04 | | 87.82 | 83.04 |
|  | #03 | 3.48 | | | 78.64 | | 68.46 | | 65.85 | | 56.74 | 46.65 |
|  | Mean | 3.12 | | | 119.96 | | 117.91 | | 116.54 | | 108.63 | 104.64 |
|  | S.D. | 2.11 | | | 43.40 | | 44.43 | | 62.45 | | 57.67 | 65.75 |
|  | SE | 0.71 | | | 14.46 | | 14.81 | | 20.81 | | 19.22 | 21.91 |
| ‡INV-001  15 mM  0.112 mg/kg | #01 | 1.19 | | | 256.04 | | 242.91 | | 231.32 | | 191.14 | 187.81 |
|  | #02 | 2.17 | | | 216.22 | | 186.95 | | 184.65 | | 181.73 | 173.97 |
|  | #03 | 4.91 | | | 125.86 | | 149.48 | | 116.53 | | 126.11 | 123.72 |
|  | Mean | 2.76 | | | 199.37 | | 193.11 | | 177.50 | | 166.32 | 161.83 |
|  | S.D. | 1.91 | | | 61.58 | | 60.92 | | 74.68 | | 39.25 | 38.03 |
|  | SE | 0.63 | | | 20.52 | | 20.31 | | 24.89 | | 16.18 | 16.81 |
| INV-001  30 mM  0.028 mg/kg | #02 | 1 | 1.98 | | 36.79 | | 39.31 | | 35.29 | | 31.02 | 30.73 |
|  |  | 2 | 1.57 | | 32.27 | | 26.44 | | 30.79 | | 32.19 | 15.46 |
|  |  | 3 | 1.48 | | 26.23 | | 26.09 | | 25.25 | | 22.57 | 13.45 |
|  | Mean | 1.67 | | | 31.76 | | 30.61 | | 30.44 | | 28.59 | 19.88 |
|  | SD | 0.26 | | | 5.29 | | 7.53 | | 5.03 | | 5.24 | 9.45 |
|  | SE | 0.15 | | | 3.05 | | 4.35 | | 2.91 | | 3.02 | 5.45 |
| INV-001  30 mM  0.056 mg/kg | #03 | 1 | 21.02 | | 62.26 | | 64.59 | | 61.47 | | 62.75 | 49.13 |
|  |  | 2 | 15.77 | | 99.98 | | 99.22 | | 38.63 | | 46.81 | 49.02 |
|  |  | 3 | 9.37 | | 126.82 | | 132.18 | | 120.21 | | 115.18 | 111.74 |
|  | Mean | 15.39 | | | 96.35 | | 98.66 | | 73.44 | | 74.91 | 69.96 |
|  | SD | 5.83 | | | 32.43 | | 33.79 | | 42.08 | | 35.77 | 36.17 |
|  | SE | 3.36 | | | 18.72 | | 19.51 | | 24.29 | | 20.65 | 20.88 |

Note: ^†^In case of administration dose of 0.028 mg/kg with a concentration of 7.5 mM, there was no visualization of lymphatic vessels except for the popliteal lymph node, and MRL images were not acquired after 50 minutes. **^‡^**Symbol indicates CNR was measured 3 times for each Beagle. CNR_LN_: contrast-to-noise ratio­_lymph node_, SD: standard deviation, SE: standard error, N: number of measurements.

**Supplementary Table 4.** Individual SNR in the lymphatic vessel

|  | Beagle  ID | N | SNR_LV_ | | | | | | | | | |
| --- | --- | --- | --- | --- | --- | --- | --- | --- | --- | --- | --- | --- |
|  |  |  | 0 min | 30 min | | 40 min | | 50 min | | 60 min | | 70 min |
| †INV-001  7.5 mM  0.028 mg/kg | #01 | 1 | 32.73 | | 60.36 | | 53.21 | | 66.56 | | N/A | N/A |
|  |  | 2 | 16.35 | | 50.15 | | 44.51 | | 47.69 | | N/A | N/A |
|  |  | 3 | 26.62 | | 31.19 | | 27.72 | | 29.01 | | N/A | N/A |
|  | Mean | 25.23 | | | 47.23 | | 41.81 | | 47.76 | | N/A | N/A |
|  | SD | 8.27 | | | 14.80 | | 12.95 | | 18.77 | | N/A | N/A |
|  | SE | 4.77 | | | 8.54 | | 7.47 | | 10.83 | | N/A | N/A |
| INV-001  15 mM  0.028 mg/kg | #01 | 1 | 65.55 | | 88.15 | | 77.39 | | 75.54 | | 104.89 | 76.54 |
|  |  | 2 | 46.58 | | 113.91 | | 149.80 | | 120.12 | | 87.11 | 105.37 |
|  |  | 3 | 54.17 | | 86.94 | | 120.82 | | 105.30 | | 79.39 | 98.84 |
|  | Mean | 55.43 | | | 96.33 | | 116.01 | | 100.32 | | 90.46 | 93.58 |
|  | SD | 9.54 | | | 15.23 | | 36.44 | | 22.71 | | 13.07 | 15.11 |
|  | SE | 5.51 | | | 8.79 | | 21.04 | | 13.10 | | 7.54 | 8.72 |
| ‡INV-001  15 mM  0.056 mg/kg | #01 | 41.15 | | | 115.06 | | 109.85 | | 121.23 | | 113.45 | 128.53 |
|  | #02 | 52.55 | | | 158.93 | | 175.31 | | 120.25 | | 122.53 | 115.01 |
|  | #03 | 36.28 | | | 127.30 | | 108.51 | | 102.75 | | 100.93 | 92.39 |
|  | Mean | 43.33 | | | 133.76 | | 131.22 | | 114.74 | | 112.31 | 111.97 |
|  | S.D. | 10.31 | | | 30.33 | | 35.54 | | 21.06 | | 15.54 | 21.71 |
|  | SE | 3.43 | | | 10.11 | | 11.84 | | 7.02 | | 5.18 | 7.23 |
| ‡INV-001  15 mM  0.112 mg/kg | #01 | 35.58 | | | 190.58 | | 161.18 | | 143.01 | | 138.59 | 135.74 |
|  | #02 | 66.74 | | | 233.14 | | 218.51 | | 228.07 | | 231.99 | 229.74 |
|  | #03 | 23.31 | | | 162.61 | | 157.80 | | 167.41 | | 163.45 | 115.73 |
|  | Mean | 41.87 | | | 195.44 | | 179.16 | | 179.49 | | 178.01 | 160.40 |
|  | S.D. | 20.13 | | | 45.26 | | 53.03 | | 66.42 | | 54.65 | 57.10 |
|  | SE | 6.71 | | | 15.08 | | 17.67 | | 22.14 | | 18.21 | 19.03 |
| INV-001  30 mM  0.028 mg/kg | #02 | 1 | 31.58 | | 42.01 | | 38.93 | | 39.46 | | 39.34 | 35.38 |
|  |  | 2 | 38.02 | | 47.21 | | 49.07 | | 44.51 | | 45.39 | 40.21 |
|  |  | 3 | 35.06 | | 45.72 | | 50.09 | | 42.92 | | 50.14 | 44.98 |
|  | Mean | 34.89 | | | 44.98 | | 46.03 | | 42.30 | | 44.96 | 40.19 |
|  | SD | 3.22 | | | 2.67 | | 6.17 | | 2.58 | | 5.41 | 4.79 |
|  | SE | 1.86 | | | 1.54 | | 3.56 | | 1.49 | | 3.12 | 2.77 |
| INV-001  30 mM  0.056 mg/kg | #03 | 1 | 29.28 | | 83.46 | | 98.47 | | 86.76 | | 105.82 | 109.31 |
|  |  | 2 | 38.95 | | 107.22 | | 135.04 | | 107.33 | | 114.07 | 105.35 |
|  |  | 3 | 45.01 | | 108.71 | | 104.55 | | 98.47 | | 97.14 | 87.62 |
|  | Mean | 37.74 | | | 99.80 | | 112.68 | | 97.52 | | 105.68 | 100.75 |
|  | SD | 7.92 | | | 14.16 | | 19.59 | | 10.31 | | 8.46 | 11.54 |
|  | SE | 4.57 | | | 8.17 | | 11.31 | | 5.95 | | 4.88 | 6.66 |

Note: ^†^In case of administration dose of 0.028 mg/kg with a concentration of 7.5 mM, there was no visualization of lymphatic vessels except for the popliteal lymph node, and MRL images were not acquired after 50 minutes. **^‡^**Symbol indicates SNR was measured 3 times for each Beagle dog. SNR_LV_: signal-to-noise ratio­_lymphatic vessel_, SD: standard deviation, SE: standard error, N: number of measurements

**Supplementary Table 5.** Individual CNR in the lymphatic vessel

|  | Beagle  ID | N | CNR_LV_ | | | | | | | | | |
| --- | --- | --- | --- | --- | --- | --- | --- | --- | --- | --- | --- | --- |
|  |  |  | 0 min | 30 min | | 40 min | | 50 min | | 60 min | | 70 min |
| †INV-001  7.5 mM  0.028 mg/kg | #01 | 1 | 1.51 | | 25.56 | | 21.96 | | 27.44 | | N/A | N/A |
|  |  | 2 | 1.35 | | 21.06 | | 18.39 | | 19.76 | | N/A | N/A |
|  |  | 3 | 3.17 | | 11.54 | | 10.34 | | 10.69 | | N/A | N/A |
|  | Mean | 2.01 | | | 19.39 | | 16.91 | | 19.31 | | N/A | N/A |
|  | SD | 1.01 | | | 7.15 | | 5.95 | | 8.38 | | N/A | N/A |
|  | SE | 0.58 | | | 4.13 | | 3.43 | | 4.84 | | N/A | N/A |
| INV-001  15 mM  0.028 mg/kg | #01 | 1 | 5.33 | | 51.11 | | 43.67 | | 41.80 | | 57.22 | 40.61 |
|  |  | 2 | 4.63 | | 62.56 | | 80.95 | | 65.91 | | 47.89 | 57.31 |
|  |  | 3 | 2.76 | | 46.41 | | 59.55 | | 55.94 | | 41.63 | 49.60 |
|  | Mean | 4.24 | | | 53.36 | | 61.39 | | 54.55 | | 48.91 | 49.17 |
|  | SD | 1.33 | | | 8.31 | | 18.71 | | 12.11 | | 7.84 | 8.35 |
|  | SE | 0.76 | | | 4.79 | | 10.80 | | 6.99 | | 4.52 | 4.82 |
| ‡INV-001  15 mM  0.056 mg/kg | #01 | 8.04 | | | 75.56 | | 71.23 | | 77.96 | | 72.45 | 83.83 |
|  | #02 | 11.08 | | | 102.56 | | 110.54 | | 69.80 | | 68.28 | 65.62 |
|  | #03 | 5.28 | | | 73.71 | | 60.51 | | 55.56 | | 53.51 | 48.32 |
|  | Mean | 8.13 | | | 83.94 | | 80.76 | | 67.77 | | 64.75 | 65.92 |
|  | S.D. | 2.92 | | | 19.43 | | 24.25 | | 13.86 | | 11.33 | 17.78 |
|  | SE | 0.97 | | | 6.47 | | 8.08 | | 4.62 | | 3.77 | 5.92 |
| ‡INV-001  15 mM  0.112 mg/kg | #01 | 3.96 | | | 144.29 | | 99.45 | | 91.93 | | 87.50 | 83.33 |
|  | #02 | 10.92 | | | 153.16 | | 144.12 | | 152.75 | | 151.62 | 150.57 |
|  | #03 | 11.11 | | | 91.13 | | 88.26 | | 84.07 | | 86.11 | 73.84 |
|  | Mean | 8.66 | | | 129.53 | | 110.61 | | 109.58 | | 108.41 | 102.58 |
|  | S.D. | 3.87 | | | 35.95 | | 37.56 | | 48.14 | | 44.01 | 43.94 |
|  | SE | 1.29 | | | 11.98 | | 12.52 | | 16.04 | | 17.96 | 17.94 |
| INV-001  30 mM  0.028 mg/kg | #02 | 1 | 6.28 | | 28.08 | | 25.86 | | 25.99 | | 26.06 | 23.20 |
|  |  | 2 | 8.11 | | 32.58 | | 33.42 | | 30.34 | | 31.04 | 27.21 |
|  |  | 3 | 7.92 | | 30.41 | | 32.96 | | 28.06 | | 32.92 | 29.31 |
|  | Mean | 7.44 | | | 30.36 | | 30.74 | | 28.13 | | 30.01 | 26.58 |
|  | SD | 1.01 | | | 2.25 | | 4.23 | | 2.17 | | 3.54 | 3.10 |
|  | SE | 0.57 | | | 1.30 | | 2.44 | | 1.25 | | 2.04 | 1.79 |
| INV-001  30 mM  0.056 mg/kg | #03 | 1 | 6.48 | | 41.54 | | 48.83 | | 42.66 | | 49.17 | 52.59 |
|  |  | 2 | 4.45 | | 56.04 | | 70.46 | | 57.10 | | 56.64 | 54.31 |
|  |  | 3 | 4.94 | | 56.89 | | 54.76 | | 50.42 | | 46.33 | 45.28 |
|  | Mean | 5.29 | | | 51.49 | | 58.01 | | 50.06 | | 50.72 | 50.72 |
|  | SD | 1.06 | | | 8.68 | | 11.17 | | 7.22 | | 5.32 | 4.79 |
|  | SE | 0.61 | | | 4.98 | | 6.45 | | 4.17 | | 3.07 | 2.76 |

Note: ^†^In case of administration dose of 0.028 mg/kg with a concentration of 7.5 mM, there was no visualization of lymphatic vessels except for the popliteal lymph node, and MRL images were not acquired after 50 minutes. **^‡^**Symbol indicates CNR was measured 3 times for each Beagle dog. CNR_LV_: Contrast-to-noise ratio­_lymphatic vessel_, SD: standard deviation, SE: standard error, N: number of measurements, N/A: not applicable

**Supplementary Table 6.** Individual T_1_, T_2_, and T_2_* relaxation time data

| Time  point | Beagle  ID | T_1_ relaxation time | | | | | T_2_ relaxation time | | | | | T_2_* relaxation time | | | | |
| --- | --- | --- | --- | --- | --- | --- | --- | --- | --- | --- | --- | --- | --- | --- | --- | --- |
|  |  | Liver^†^ | Right Kidney^†^ | | Left kidney^†^ | | Liver^†^ | Right kidney^†^ | | Left kidney^†^ | | Liver**‡** | Right kidney^†^ | | Left kidney^†^ | |
|  |  |  | Cor | Med | Cor | Med |  | Cor | Med | Cor | Med |  | Cor | Med | Cor | Med |
| 0 h | #01 | 328.12 | 445.48 | 571.76 | 516.86 | 602.97 | 49.91 | 111.85 | 154.21 | 96.41 | 153.17 | 18.47 | 27.26 | 37.78 | 24.96 | 38.44 |
|  | #02 | 331.41 | 459.54 | 572.37 | 530.97 | 575.57 | 47.74 | 120.29 | 183.47 | 104.32 | 167.21 | 12.75 | 24.31 | 36.24 | 24.51 | 36.96 |
|  | #03 | 324.24 | 451.75 | 569.37 | 535.37 | 591.65 | 46.36 | 114.17 | 156.42 | 94.38 | 146.35 | 14.32 | 17.95 | 24.88 | 21.93 | 33.75 |
|  | Mean | 328.27 | 452.25 | 571.17 | 527.73 | 590.06 | 48.01 | 115.43 | 164.70 | 98.37 | 155.58 | 15.18 | 23.17 | 32.97 | 23.80 | 36.38 |
|  | SD | 20.41 | 31.16 | 23.21 | 30.77 | 38.35 | 1.67 | 4.99 | 14.50 | 6.43 | 10.33 | 2.61 | 4.89 | 6.51 | 3.71 | 3.54 |
|  | SE | 6.45 | 9.85 | 7.34 | 9.73 | 12.12 | 0.55 | 1.44 | 4.18 | 1.85 | 2.98 | 0.67 | 1.41 | 1.88 | 1.07 | 1.02 |
| 48 h | #01 | 287.73 | 441.45 | 616.97 | 526.01 | 660.40 | 47.80 | 110.31 | 144.68 | 104.70 | 146.36 | 16.21 | 27.24 | 38.53 | 29.15 | 40.01 |
|  | #02 | 300.67 | 466.95 | 587.58 | 525.41 | 598.33 | 47.86 | 117.46 | 180.09 | 105.63 | 178.58 | 12.27 | 22.98 | 26.47 | 25.22 | 37.89 |
|  | #03 | 357.43 | 470.01 | 600.28 | 511.42 | 591.31 | 45.95 | 110.08 | 155.69 | 96.65 | 147.86 | 10.98 | 17.69 | 30.23 | 23.11 | 35.24 |
|  | Mean | 313.82 | 459.47 | 601.61 | 520.95 | 616.68 | 47.21 | 112.62 | 160.15 | 102.33 | 157.60 | 13.15 | 22.64 | 31.74 | 25.82 | 37.71 |
|  | SD | 42.49 | 27.39 | 35.21 | 21.55 | 60.02 | 1.24 | 4.98 | 16.71 | 6.23 | 16.85 | 2.37 | 6.47 | 8.89 | 3.32 | 3.69 |
|  | SE | 13.43 | 8.66 | 11.13 | 6.81 | 18.98 | 0.41 | 1.43 | 4.82 | 1.81 | 4.86 | 0.61 | 1.86 | 2.56 | 0.96 | 1.06 |

**Note**: T_1_, T_2_, and T_2_* maps were measured with three times for each beagle. SD: standard deviation, SE: standard error, Cor: cortex, Med: medulla. ^†^symbol means no statistically significant differences in before and after administration (*p* > 0.05). On the other hand, **^‡^**symbol indicates statistically significant difference in before and after administration (*p* < 0.05).
